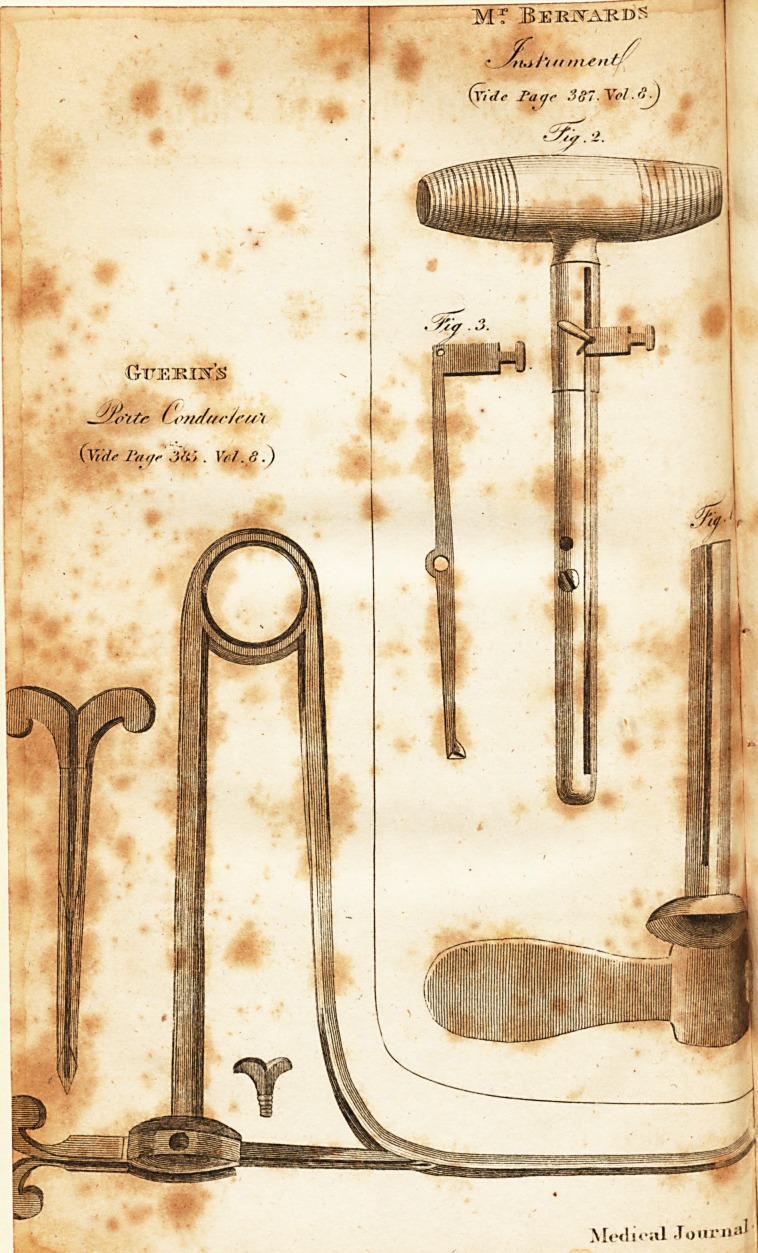# Of the Cure of an Ovarian Dropsy, Where the Cyst Is Single, by Means of Incision in the Cyst; Thereby Procuring a Similarly Favourable Event in All Such Cases, to That Which an Accidental Rupture of the Cyst Sometimes Produces; with a Plate, Describing the Instrument for Making the Incision

**Published:** 1802-11-01

**Authors:** 

**Affiliations:** Southampton


*Of* THE CURE OF AN OVARIAN DROPSY, *where the Cyst is single, by means of Incision in the Cyst; thereby procuring a similarly favourable Event in all such Cases, to that which an accidental Rupture of the Cyst sometimes produces; with a Plate, describing the Instrument for making the Incision; communicated by Mr.* BERNARD, *of Southampton.*

IN the number of thofe difeafes which the fkill of the Phyfician or the hand of the Surgeon is not able to cure, may be counted thofe dropfies peculiar to females which originate in the appendages of the uterus. Thefe dropfies are not unfrequent. They may be divided into thofe in which the cyfts are fingle, or in which they are more in number, accompanied with hydatids more or lefs in quantity, and thofe where the parts are enlarged fo as to be an infeparable mafs of tumours.

Of female dropfies, cafes may be concluded to be proper for the operation propofed if no very confiderable tumour or induration be found after the firft tapping, on examining the abdomen.

In order to favor the cure, it is to be wifhed that the firft operation be performed before the enlargement is to a great degree. It is defirable alfo that the fecond tapping, accompanied with the operation of dividing the cyft, fhould be performed in a ftate of moderate diftenfion, in order that the fac in a collapfed ftate, which the operation will reduce it to, may be lefs inconvenient in proportion to its fize.

The operation propofed, is, to make ufe of the common trocar with a canula adapted to it, in which canula there is to be a flit or groove, fig. 1. Immediately after puncturing with the common trocar it is to be withdrawn, and before the exit of any confiderable portion of the fluid, a blunt trocar is to be introduced, fig. 2, in which alfo is a flit or goove correfponding with that in the canula. In this groove is placed a biftourie cachée, fig. 3, turning on its centre, in order to deprefs or elevate a lancet point through the opening in the canula to the height of one-twelfth of an inch or thereabout from the furface of the canula. The lancet point being depreffed in the groove for introduction, the operator changes the pofition of the inftrument thus introduced through the abdominal coverings and fac from their courfe or direction, at firft to a pofition which fhall make the handle of the inftrument lie in contact with the fkin, and fo that the groove or flit fhall be in a line with the furface of the abdominal coverings, fo that in immediate contact with the groove in the canula is the fac interpofed betwixt it and the abdominal coverings, which by the hand of an affiftant ant are to be preffed fo as to fix more firmly the groove in contact with the fac. The lancet point being elevated by the thumb of the operator (by which it can be raifed or depreffed) the blunt trocar containing the lancet, is to be withdrawn, to make in its courfe an incifion of the length of the flit, about two inches in the cyft. The lancet point, when it has gone the length of the flit, muft be again depreffed to be withdrawn.

The operator may after this permit the flow of more or lefs of the fluid as he judges proper. I fhould think a portion of it may as well be detained.

With refpect to the fuccefs of the operation, my idea is, that the lips of this incifion can never again come in contact with each other, fo as to unite and detain the fecreted fluid, which being therefore admitted into the cavity in which the cyft is contained, will be taken up by its abforbents, and the patient be exempted from immenfe accumulation, or repeated tappings, from either of which death follows in a few years.

I have compleated in my own mind for a long time, and provided myfelf with the inftrument above defcribed, being fatisfied that it is a preferable method to any hitherto propofed, whether by injecting a fluid into the cavity or introducing a feton.

In order to obtain additional information in the proportion of patients affected with the difeafe of fingle cyft, I have of late years made inquiry, efpecially in London, refpecting dropfies peculiar to females, among gentlemen of the profeffion, (to whom I would exprefs my acknowledgements) in order to afcertain the propriety of communicating my method. About thirty years ago, I printed and difperfed a feries of queftions addreffed to the Faculty refpecting the various kinds of dropfies, in the form of a letter, leaving room in the faid letter for the infertion of anfwers. Being now arrived at feventy years, I think further inquiry will be precluded me, I therefore leave the above ftatement, hoping it may be brought into ufe in fuch cafes, and be attended with fuccefs.

## Figures and Tables

**Fig. 3. Fig. 2. Fig. 1 f1:**